# I-PREGNO – prevention of unhealthy weight gain and psychosocial stress in families during pregnancy and postpartum using an mHealth enhanced intervention: a study protocol of two cluster randomized controlled trials

**DOI:** 10.1186/s12884-023-05735-x

**Published:** 2023-06-06

**Authors:** Lea Vogel, Tanja Färber, Ingrid Hölzl, Tom Deliens, Carmen Henning, Christoph Liel, Johanna Löchner, Ulrike Lux, Ansgar Opitz, Caroline Seiferth, Vicka Versele, Jörg Wolstein, Mireille N. M. van Poppel

**Affiliations:** 1 Department of Psychology, LMU Munich, Munich, Germany; 2 National Center for Early Prevention, German Youth Institute, Department of Families and Family Policies, Munich, Germany; 3 Department of Pathopsychology, University of Bamberg, Bamberg, Germany; 4 Institute of Human Movement Science, Sport and Health, University of Graz, Graz, Austria; 5 Department of Movement and Sport Sciences, Faculty of Physical Education and Physiotherapy, Vrije Universiteit Brussel, Brussels, Belgium; 6 Department of Child and Adolescent Psychiatry, Psychosomatics and Psychotherapy, University Hospital of Psychiatry and Psychotherapy Tuebingen, Tuebingen, Germany; 7 Faculty of Medicine, Department of Development and Regeneration, KU Leuven, Herestraat 49, 3000 Leuven, Belgium

**Keywords:** mHealth, Pregnancy, Gestational weight, Unhealthy weight gain, Weight retention, Postpartum, Psychosocial burden, Blended counseling, cRCT

## Abstract

**Background:**

The transition to parenthood represents a critical life period with psychosocial, and behavioral changes and challenges for parents. This often increases stress and leads to unhealthy weight gain in families, especially in psychosocially burdened families. Although universal and selective prevention programs are offered to families, specific support often fails to reach psychosocially burdened families. Digital technologies are a chance to overcome this problem by enabling a low-threshold access for parents in need. However, there is currently a lack of smartphone-based interventions that are tailored to the needs of psychosocially burdened families.

**Aims:**

The research project I-PREGNO aims to develop and evaluate a self-guided, smartphone-based intervention in combination with face-to-face counseling delivered by healthcare professionals for the prevention of unhealthy weight gain and psychosocial problems. The intervention is specifically tailored to the needs of psychosocially burdened families during the pregnancy and postpartum period.

**Methods:**

In two cluster randomized controlled trials in Germany and Austria (N = 400) psychosocially burdened families will be recruited and randomized to i) treatment as usual (TAU), or ii) I-PREGNO intervention (self-guided I-PREGNO app with counseling sessions) and TAU. We expect higher acceptance and better outcomes on parental weight gain and psychosocial stress in the intervention group.

**Discussion:**

The intervention offers a low cost and low-threshold intervention and considers the life situation of psychosocially burdened families who are a neglected group in traditional prevention programs. After positive evaluation, the intervention may easily be implemented in existing perinatal care structures in European countries such as Germany and Austria.

**Trial registration:**

Both trials were registered prospectively at the German Clinical Trials Register (Germany: DRKS00029673; Austria: DRKS00029934) in July and August 2022.

## Background

The perinatal period represents a critical period for the families’ health with biological, psychosocial, and behavioral changes and challenges. Due to health behavior changes (including changes in eating behavior, sleep behavior, physical activity and sedentary behavior) the transition to parenthood represents an inflection point for overweight and obesity, such that perinatal weight gains are retained in the long term [1, 2]. In Europe, three out of every four pregnant women gain more weight than recommended by the Institute of Medicine [3, 4]. Although many mothers loose this extra weight again at 12 months postpartum, about 20 – 50 % of women still face postpartum weight retention which contributes considerably to their risk of developing overweight and obesity [5, 6]. Perinatal unhealthy weight gain can also be observed in fathers [7]. Recent findings show that also fathers gain a significant amount of weight and fat mass during pregnancy, with some even gaining almost 10 kg [7, 8]. Maternal excessive gestational weight gain moreover has a negative influence on their offspring [9–11]. In addition, the postpartum period is also crucial for the offspring’s risk of developing chronic diseases, including obesity. The children’s risk for overweight and obesity depends on parental feeding practices, well-being and the mother-child relationship [12].

The main modifiable health or lifestyle behaviors contributing to unhealthy weight gain are dietary intake and physical activity [13]. Women and men experience different changes in dietary intake and physical activity throughout the perinatal period which can be explained by determinants at the individual, interpersonal, environmental and policy level [14–16]. Hence, existing gestational weight gain interventions often focus on eating behavior or physical activity [17]. Studies that have investigated both behaviors during the perinatal period show that psychological and social factors (e.g. emotion regulation, depression, social support) influence these lifestyle behaviors, especially during the perinatal period [18, 19]. In prevention programs that target pregnant women in order to prevent unhealthy perinatal weight gain, the underlying psychological factors have been barely considered [20]. Since the transition to parenthood represents a period in which parents often experience heightened psychosocial stress and symptoms of depression, sleeping disorders and anxiety [1], integrating strategies that promote the parental well-being and prevent psychosocial stress could increase the effectiveness of perinatal weight management interventions.

Psychosocially burdened families (e.g., families with low socio-economic status (SES), mental health problems) are particularly vulnerable to unhealthy gestational weight gain and weight retention after birth [21–24]. Yet, there is no monocausal pathway to unhealthy weight gain or health impairments in general, but socioeconomic burden is often linked to impaired health via cumulative risk exposure and poor health behavior or literacy [25–29]. Besides socioeconomic burden, also other psychosocial burden e g., depression, relationship distress or ineffective emotion regulation are considered as correlates or mediating mechanisms for unhealthy weight gain or overweight [30, 31]. However, little is known about the effectiveness of preventive interventions among these vulnerable groups since those families are often not reached by prevention programs and interventions – not even by digital ones [32–34]. Furthermore, the specific needs of psychosocially burdened families are often not taken into account in the development of new interventions. Hence, there is a clear need for preventive interventions that are specifically tailored to the needs and life situations of this vulnerable group.

In recent years, in the hope of reaching more individuals, especially those who cannot be reached through traditional counseling approaches, the development and implementation of mobile health (mHealth) interventions has emerged. Apps provide a low-threshold and inexpensive access, and could represent an added value to existing programs especially for time-restricted phases of life such as the pregnancy or perinatal period [35–37]. Today, almost everybody in Europe has a smartphone regardless of SES. Especially the current generation of expectant parents is familiar with digital tools and the acceptance rates of mHealth is extremely high compared to other target groups [38]. Hence, an app-based prevention approach is promising to reach the neglected group of psychosocially burdened families in prevention services. Furthermore, mHealth interventions seem to be even more promising if they are combined with personal contacts [39]. As preceding research suggests, an mHealth intervention tailored to individual needs in combination with successful face-to-face counseling might be particularly useful for reaching people with low SES [40, 41]. Despite such advantages of low-threshold supply of mHealth interventions, many recent lifestyle counseling and smartphone-based weight management interventions did not include individuals with psychosocial burdens in either pregnancy or the postpartum period [40, 42]. In addition, the majority of such interventions neglect fathers (to-be) and have a strong focus on maternal needs [40]. However, paternal involvement may increase intervention effects substantially by providing additional social and practical support for mothers (and the family) buffering the experienced stress during this challenging and vulnerable period of life [43].

Based on the challenges during COVID-19 and the fast-evolving digital change in the last decade, an mHealth based intervention could be an effective approach to prevent unhealthy weight gain and psychosocial stress in vulnerable families during the perinatal period. The intervention should initially focus on psychological factors known to be related to health behaviors affecting the perinatal weight gain, involve both parents, and should be extended over the complete perinatal period. As first of its kind, the overall objective of I-PREGNO is the development and evaluation of an mHealth intervention that aims to prevent unhealthy weight in psychosocially burdened families through the promotion of health behavior (i.e., eating behavior and physical activity), and the parental well-being during the perinatal period. I-PREGNO was developed from June 2021 to July 2022 and consists of a self-guided multicomponent smartphone app combined with face-to-face counseling that can be implemented into existing perinatal care services. In two cluster randomized controlled trials (cRCTs) we will examine the efficacy of the intervention in existing health and family care services during pregnancy and the postpartum period. We expect that our developed mHealth intervention has a positive effect on the prevention of unhealthy weight gain (indicated by a body mass index (BMI) that is more stable than in the control condition) during the pregnancy and postpartum period and promotes psychological competences (i.e., emotion regulation, self-efficacy) that reduce stress and poor mental health particularly in vulnerable families.

## Methods

The study design is reported in line with the SPIRIT 2013 Statement (Standard Protocol Items: Recommendations for Interventional Trials) [44]. The study has received ethical approval from the ethical committee of the University in Bamberg (nr. 2022-02/09) and of the Medical University in Graz (nr. 34-249 ex21/22). Both trials were registered in the German register for clinical trials (DRKS00029673; DRKS00029934).

### Overall study design

**Fig. 1:**
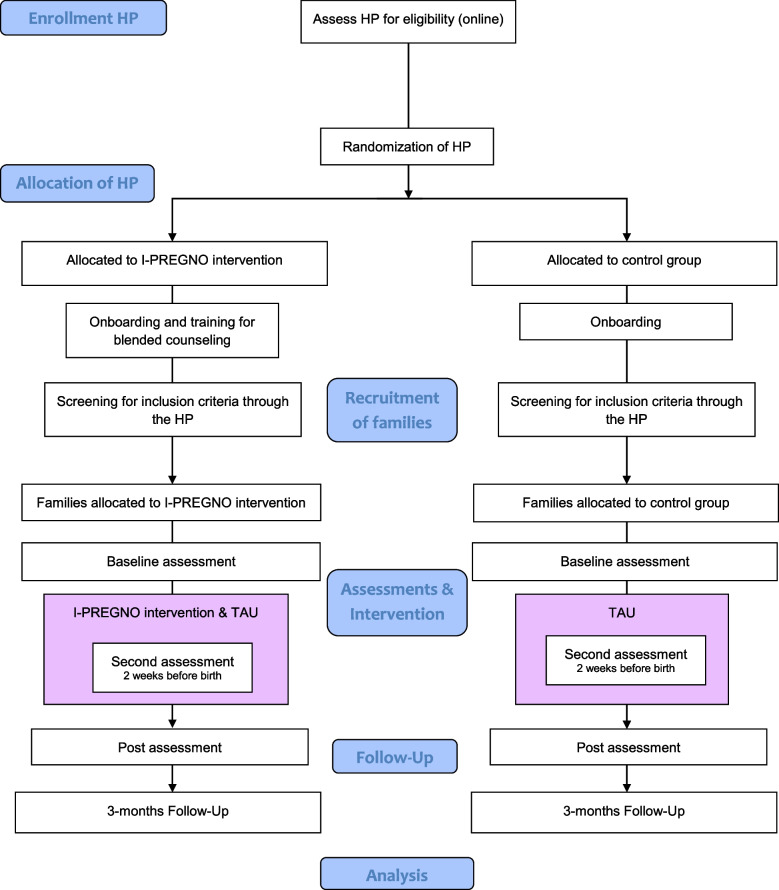
CONSORT Flowchart for the trial in Austria during pregnancy and postpartum *Note.* HP = Healthcare professionals (midwives); TAU = treatment as usual

**Fig. 2:**
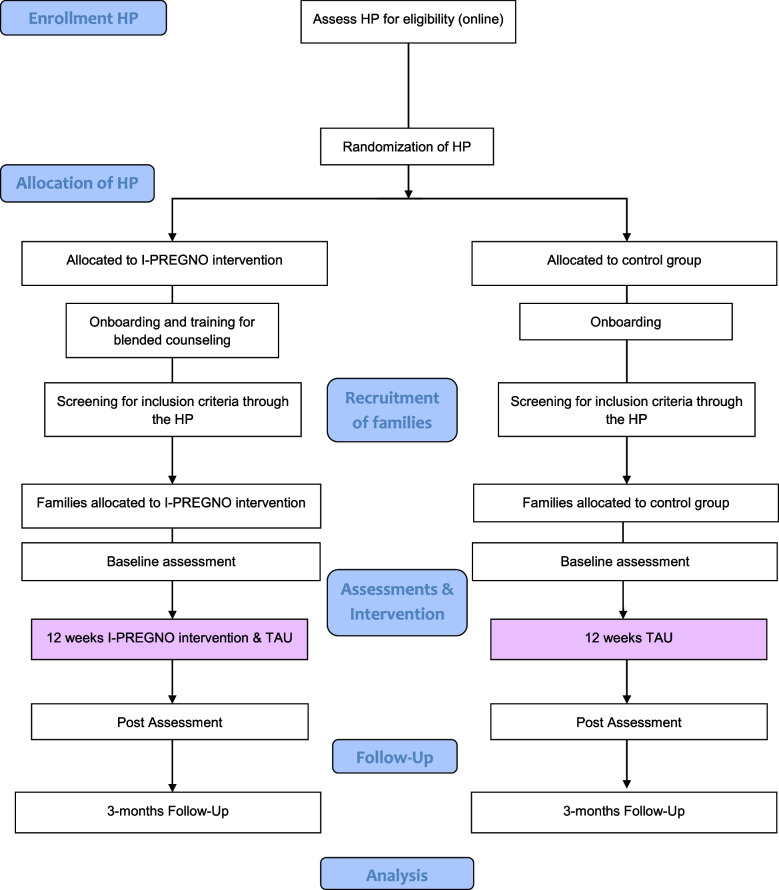
CONSORT flowchart for the trial in Germany during the postpartum period *Note.* HP = Healthcare professionals (family midwives, family nurses and social workers); TAU = treatment as usual

I-PREGNO is a multinational study, with two study centers in Germany and one study center in Austria. The trial’s design is a multicenter cRCT aiming to reduce the risk of unhealthy weight gain and promote the well-being of vulnerable families during the perinatal period. Due to the different healthcare structures, the study designs in both countries differ in some characteristics. Therefore, some parts of the trials are described separately in the following text. Figures 1 and 2 provide an overview of the study trials.

### Setting

We will use a parallel two-arm cRCT design with one intervention group, receiving the I-PREGNO blended counseling intervention plus treatment as usual (TAU) and one control group with TAU only. *In Austria*, the trial will start during the second trimester of pregnancy and continue until the postpartum period. As we focus on psychosocially burdened families, our sample will be recruited mainly by midwives who have a contract with public healthcare operators. In *Germany*, we will investigate the efficacy during the first year postpartum. Our target group will be recruited within a nationwide *Early Childhood Intervention* program, particularly a selective prevention home-visiting program for psychosocially burdened families delivered by midwives and nurses with a special training, and in some federal states or municipalities also other professionals. All professionals are summarized under the term healthcare professionals (HPs) in the following text.

### Recruitment of Healthcare Professionals

HPs are supposed to recruit participants and conduct the blended counseling of the intervention in both countries. In *Austria*, the eligibility criteria of HPs are being a certified midwife within the Austrian public healthcare system, offering a counseling session during pregnancy and the postpartum period, and sufficient knowledge of the German language. To motivate HPs to participate in our study, we have worked closely with the clinical Department for Obstetrics and Gynaecology in Graz (Austria) as well as the midwifery committee of Austria. The study team of Graz will send advertising emails and conduct cold calls to promote the project.

In *Germany,* HPs within the national early childhood intervention program represent all individuals who are either in training or already have a degree as a family midwife or family nurse. Eligibility criteria are the support of at least one family who can be included in the study and sufficient German language skills. To motivate HPs to participate in our study, we have worked closely with the program’s coordination units in different federal states in Germany. They will promote I-PREGNO and will send advertising emails to all HPs in their state. In addition, the study team will offer online information events in which the intervention and the process of the study will be presented. Participating HPs in both countries will receive €50 for every family they recruit and that completes the baseline assessment.

### Participants

For a family to participate, at least the mother has to agree to participate in the study. If the father of a family is also present and agrees to participate, he can be registered separately by the HP. Families will be enrolled by the HP once the mother agrees to participate in the study.

*In Austria,* eligible families should a) meet one of the criteria of low SES (defined as: no partner or receipt of financial benefits or low education), b) expect a child (gestational age between the 18^th^ and 22^th^ week) and c) own a smartphone. *In Germany,* eligible families are those who a) meet at least one of the psychosocial burden factors (see Table 1), b) have a child at the age between 0-12 months, c) own a smartphone, and d) receive home-visiting support from a HP for a period of at least 12 weeks.

**Table 1:** List of psychosocial burden factors as part for the inclusion criteria in Germany

**Psychosocial burden factors**	• financial problems • single parenthood • mental illness of one parent • underaged mother • insecure living situation • traumatic life events in the past • social isolation / lack of integration • birth of multiples and rapid succession of births • premature birth of the child • regulatory disorder of the child • chronical illness, developmental delay and/or disability of the child • relationship distress

In both countries, families will be excluded from the study if they meet at least one of the following exclusion criteria: a) mother is younger than 16 years; b) acute mental health problems that hamper the ability to participate (e.g., suicidality, mania); c) chronic disease that can influence behavior related to energy balance (diabetes, pre-eclampsia, etc.) or require a complex diet, d) insufficient knowledge of the German language (which does not allow the use of the app and the completion of the questionnaires) or e) other reasons that do not allow the correct use of the app, counseling or completion of the questionnaires. Each family will receive €50 for participation if all questionnaires are completed.

### Sample size calculation

For the primary outcome (weight), we have calculated in G*Power that we need to recruit 100 families in each study arm, based on two-sided testing with alpha of 5%, and assuming 20% drop out in order to have at least 80% power to detect a 2 kg difference in weight gain/retention between intervention arms. This is based on the intervention effect on gestational weight gain of the DALI intervention (Simmons D: JCEM 2016), and assuming non-significant clustering in weight within HPs. With this sample size, a 0.45 (SD 1.0) difference in birthweight z-score or infant BMI z-score can be found with 80% power. Since both trials will initially be analyzed separately and assuming non-significant clustering in weight within HPs, clustering by country (Germany, Austria) is not considered in the sample size calculation. Based on previous exchange with HPs, we estimated 2-4 families per HP as realistic. Therefore we intend to recruit at least 60 HPs in each study arm.

### Procedure

In *Austria,* HPs will register online and complete an online questionnaire including the consent to the study as well as questions about their professional background. After registration, they will be randomized to one of the study arms (I-PREGNO combined with TAU versus TAU only). Afterwards the study coordinator will get in contact with the HPs via telephone (onboarding). During the telephone call, the HPs will be informed about the condition they have been randomly assigned to and will receive a 20-30 minute briefing with instructions on the study materials. Before they start the recruitment of families, HPs who have been assigned to the intervention condition will receive a five-hour-online-training for the blended counseling. In a second step, the HPs will start to inform every pregnant woman, who has an appointment for the first counseling (between week 18-22 of pregnancy), and send the registration link to interested families. Interested families will also receive information material and data protection information. After the registration, families that meet the eligibility criteria will receive a link for the online baseline questionnaire (t0) from the study team, which has to be completed before the appointment with their midwife. After the families have completed the baseline assessment, the HP and participants receive a message to start the intervention phase and, in case of the intervention condition, a download guide for the app. Five weeks before birth, families receive the link for a second assessment (t0.5) via email. Eight weeks after birth, the families will receive the link for the post assessment (t1), which marks the end of the intervention. Three months after the end of the intervention, participants will be asked to complete a follow-up online questionnaire (t2). In case of non-response, participants will be reminded via email and telephone through the I-PREGNO coordination team.

In *Germany*, HPs will be able to register online and complete an online questionnaire containing the consent to the study, the screening for inclusion and exclusion criteria, as well as questions about their professional background. Eligible HPs will be randomized to one of the two study conditions, contacted via email with the information about their group assignment, and sent forms and materials by mail. Afterwards, the study coordination will get in contact with the HPs via telephone and conduct a 20-30 minutes briefing with instructions on the study materials (onboarding). If they have been assigned to the intervention condition, they will receive a five-hour online training for the blended counseling. In a second step, the HPs will screen each of their families for the eligibility criteria, inform potential families about the study, and provide information material, as well as data protection policies to interested families. HPs will be able to register families online. The participating families receive an individualized link for the baseline assessment (t0) via mail. After the families have completed the assessment, the HP and the participants receive a message to start the intervention and – in case of the intervention condition – a download guide for the app. After 12 weeks, the families receive the link for the post assessment (t1) via email. Three months after the end of the intervention, participants will be asked to complete a follow-up online questionnaire (t2). In case of non-response, participants will be reminded via email and telephone as well as through the HP. In both countries, the app access will be deactivated after the intervention period for the intervention group and (again) activated for each participant (also for the control group) after the last assessment (t2) is completed.

### Randomization

Cluster randomization was chosen to prevent contamination of the different conditions. This means that every HP should provide the same treatment to all of their families participating in the I-PREGNO study. Participating HPs will be randomly assigned to one of the two study arms and will be informed about the assignment immediately. The randomization will be performed using a computerized random number generator, pre-stratified for the two trials provided by an external, independent expert from the Department of Computing in the Cultural Sciences at the University of Bamberg.

### Interventions

#### I-PREGNO

The I-PREGNO intervention follows a blended counseling approach, combining a self-guided app for families with face-to-face counseling conducted through the HPs. The interventions will be implemented in existing healthcare structures in Germany and Austria.

The interactive self-guided modular I-PREGNO app, integrates basic principles of evidence-based cognitive-behavioral therapy (CBT) and behavior change techniques [45]. The app aims to help families to increase their mental health, to reduce psychosocial stress, and to broaden their knowledge about nutrition and physical activity topics via the completion of a variety of thematic modules. I-PREGNO was built on the technical and content-related knowledge gained from the gender-sensitive multicomponent mHealth weight loss intervention I-GENDO [46]. Both apps were developed within an iterative process by the authors working closely with an external software provider (groupXS Solutions GmbH). To address the needs of psychosocially burdened families and to adapt the existing content and approach of the intervention, we conducted focus groups with families and HPs (midwives, family midwives, health nurses, social workers).

**Fig. 3:**
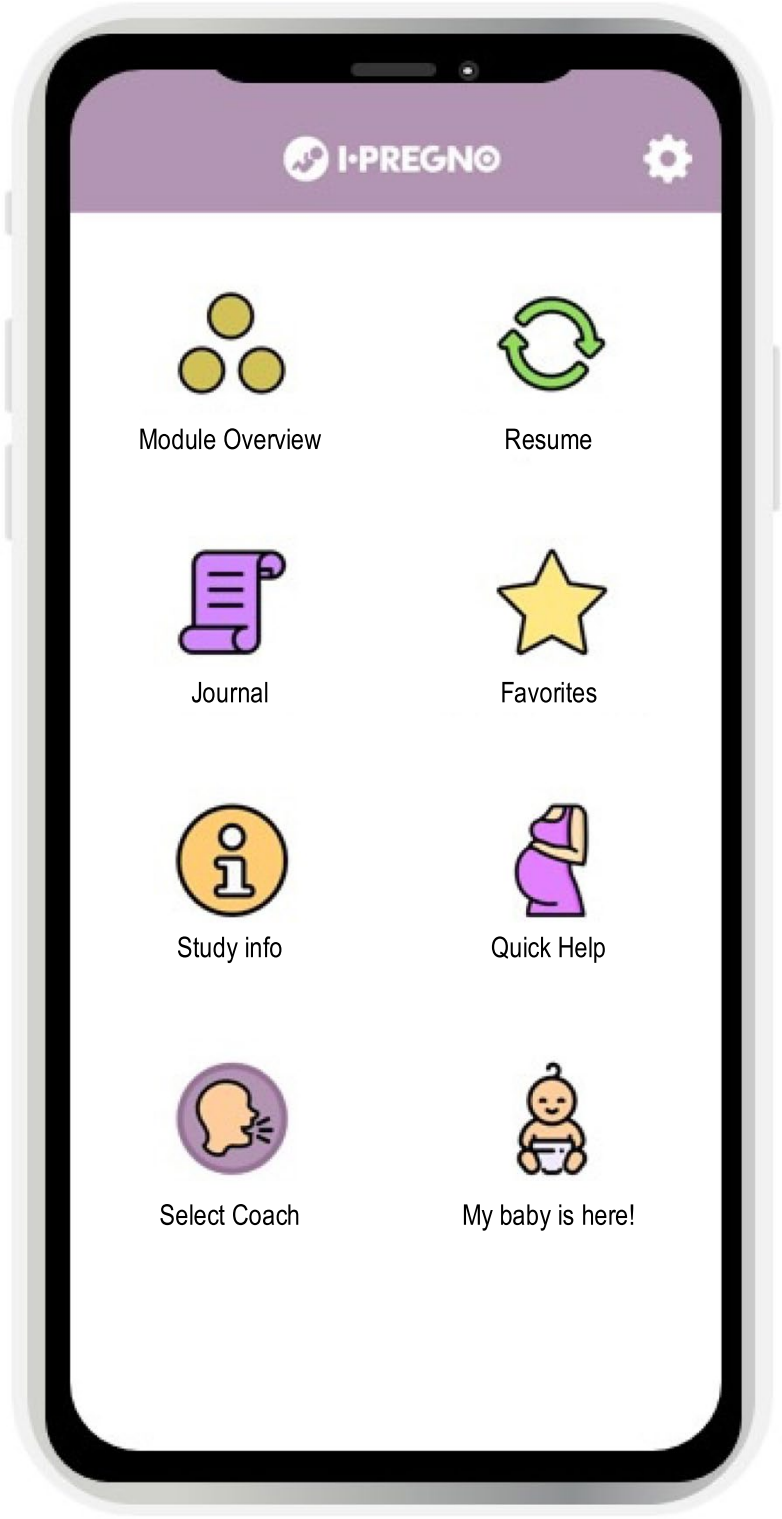
Interface of the App I-PREGNO

Figure 3 provides an overview of the I-PREGNO app interface. The app consists of a modular psychological intervention (‘Module Overview’, ‘Resume’), self-monitoring of mood, exercise eating behavior, and sleep quality through a personalized diary (‘Journal’), a folder to save favorite exercises and strategies (‘Favorites’), information about the study itself and emergency contacts (‘Study Info’, ‘Quick Help’), and a selection of accompanying coaches (‘Select Coach’). One particularity is the possibility to adapt the content to the phase the user is currently in (pregnancy or postpartum; “My baby is here!”). In addition to this individual customization within the app, we provided two app releases with slightly adapted content, one for the mothers and one for the fathers to ensure that the app content fits to the different needs and perspectives. The app is available for smartphones with iOS and Android. To use all app features a study code and password is required. The core of the app, the psychological intervention, includes 12 modules that focus on improving specific psychosocial or behavioral aspects associated with gestational weight gain, retention, and perinatal mental health. Two modules address the introduction to (getting to know the app, goal setting) and one addresses the conclusion (review and relapse prevention) of the intervention program. Seven more modules focus on improving core competences for parents: self-care, stress management skills, emotion regulation skills, self-efficacy, self-esteem, mindfulness, and social competences/social support. The remaining two modules address nutrition and exercise behavior of parents. These have been created within the framework of the counseling and contain appropriate exercises and information.

Each module consists of a various number of sessions (range: 2 - 18). To ensure autonomy, users are able to work through the modules and sessions as desired. In line with existing research [47], we recommend users to use the app at least three times a week with a duration of approximately 15 minutes to improve the targeted core competences. Each completed session is marked with a check mark and the module tracks the user’s progress. The sessions include psychoeducational information, several CBT-based self-help techniques, skill trainings (i.e., coping strategies, cognitive restructuring) and, a range of behavior change techniques (i.e., goal setting, monitoring, shaping knowledge) delivered through text, audio, images, and interactive elements that require participants’ input (e.g., filling out gap texts).

The face-to-face counseling includes guidance for health behavior change focusing on physical activity and nutrition and is delivered by the HP. It was built on principles of patient empowerment and CBT skills inspired by Motivational Interviewing [48]. The counseling is based on seven key messages (*[1]Make water YOUR drink of choice, [2] Diversity does not have to be a lot, [3] Colorful and healthy: plant-based foods, [4] Snacks & fast food: (un)canny seducers, [5] Finding the right amount of meat, [6] Finding joy in movement and sitting less, [7] Train your strength*) that are also presented within the app. The counseling was developed together with HP so that it can easily be implemented in the existing face-to-face appointments during prenatal care and early intervention programs after birth. During the counseling, specific messages will be chosen together with the HP, depending on the knowledge as well as individual needs of the families. In our study, HPs will integrate the counseling in the appointments of TAU with their families (on average five sessions). The counseling takes about 10-15 minutes per appointment. 

The HPs in the intervention group will be trained in the I-PREGNO key messages (i.e., psychoeducation on eating and physical activity behavior) and in Motivational Interviewing techniques (i.e., ways to address the key lifestyle messages wih families, approaches to work through resistance). Sports scientists, psychologists and an ecothrophologist with further qualifications in Motivational Interviewing will deliver the training. HPs will be supervised by a Motivational Interviewing expert throughout the course of the intervention phase.

#### Treatment as usual

In both countries, both groups will receive TAU. In *Austria,* TAU represents perinatal care delivered by midwives (with usually one appointment before and up to seven appointments after birth). During the first appointment, usually scheduled at 18-22 weeks of gestation, topics such as the place of birth, nutrition and physical activity during pregnancy are discussed, as well as information on health promotion and preventive behavior. From the first to the fifth day after birth, the midwife will check on the mother and baby once a day. From the sixth day up until eight weeks after birth, women have up to seven further optional appointments with the midwife. During the appointments, medical examinations are carried out and information on health behavior (including physical activity and nutrition), and breastfeeding is provided.

TAU in *Germany* represents a long-term home-visiting program for psychosocially burdened families within the framework of early childhood intervention that usually starts during the postpartum period. The preventive and voluntary offer is particularly tailored to families who find themselves in psychosocially burdening living conditions. The service is delivered by trained HPs (family midwives, family and child nurses) and thus aims to have a low-threshold access. Regular home visits (usually weekly) are intended to provide comprehensive support regarding questions about child development or coping with everyday tasks for psychosocially burdened families with children aged between zero and three years. It is provided nationwide but with local configurations. The home-visiting program is funded by the Federal Ministry of Family Affairs, Senior Citizens, Women and Youth (BMFSFJ) as part of the Federal Foundation for Early Childhood Intervention (Bundesstiftung Fruhe Hilfen).

### Outcomes

Our primary outcome will be body composition measured through body mass index (BMI) of the family members (self-reported, the mother’s, if available father’s and the child’s height and weight). Secondary outcomes will be eating behavior (i.e., eating styles), physical activity, and psychological factors (i.e., depression, emotion regulation, parental stress) that are associated with the family’s well-being and influence eating behavior and physical activity. Information about the children (height, weight, breastfeeding) is collected through the mothers. Since the project primarily intends to reach psychosocially burdened families another focus lies on socio-demographic characteristics and socio-economic stressors (e.g., whether families receive social support, health status, crowded living conditions, and the migrant status of the parents). Data will be gathered at three (Germany) or four (Austria) time points. Tables 2 and 3 provide an overview of the included measurements.

**Table 2:** Data collection at different time points during the study in Austria

Questionnaire	t_0_	t_0.5_	t_1_	t_2_
	18^th^ to 22^nd^ week of pregnancy	35^th^ -37^th^ week of pregnancy	8 weeks pp	3 months after t_1_
Socio-demographics	x			
Weight of the parent	x	x	x	x
Child’s weight and height			x	x
DEBQ	x	x	x	x
FFQ	x	x	x	x
IPAQ	x	x	x	x
EPDS	x	x	x	x
DERS-SF	x	x	x	x
ECR-RD		x		
GSE	x	x	x	x
CTS		x		
PSI-SF			x	x
PBQ			x	
I-FEEL				x
Regulatory Disorders				x
MAUQ			x	

*DEBQ* Dutch Eating Behaviour Questionnaire, *FFQ* Food Frequency Questionnaire, *IPAQ* International Physical Activity Questionnaire, *EPDS* Edinburgh-Postnatal-Depression-Scale, *DERS-SF* Difficulties in Emotion Regulation Scale, *ECR-RD* Experiences in Close Relationships, *GSE* General Self-Efficacy Scale, *CTS* Childhood Trauma Screener, *PSI-SF* Parenting Stress Index, *PBQ* Postpartum Bonding Questionnaire, *MAUQ* mHealth app usability questionnaire, *pp* postpartum

**Table 3:** Data collection at different time points during the study in Germany

Questionnaire	t_0_	t_1_	t_2_
	0-12 months pp	3-15 months pp	3 months after t1
Socio-demographics	x		
Weight of the parent	x	x	x
Child’s weight and height	x	x	x
DEBQ	x	x	x
FFQ	x	x	x
IPAQ	x	x	x
EPDS	x	x	x
DERS-SF	x	x	x
ECR-RD		x	
GSE	x	x	x
CTS	x		
PSI-SF	x	x	x
PBQ	x	x	x
I-FEEL			x
Regulatory Disorders			x
MAUQ		x	

*DEBQ* Dutch Eating Behaviour Questionnaire, *FFQ* Food Frequency Questionnaire, *IPAQ* International Physical Activity Questionnaire, *EPDS* Edinburgh-Postnatal-Depression-Scale, *DERS-SF* Difficulties in Emotion Regulation Scale, *ECR-RD* Experiences in Close Relationships, *GSE* General Self-Efficacy Scale, *CTS* Childhood Trauma Screener, *PSI-SF* Parenting Stress Index, *PBQ* Postpartum Bonding Questionnaire, *MAUQ* mHealth app usability questionnaire, *pp* postpartum

#### Eating style

To assess eating styles, the German version of the Dutch Eating Behavior Questionnaire - DEBQ [49–51] will be used. The DEBQ is a questionnaire consisting of 30 items with three different subscales for the eating styles: *Emotional eating* (eating due to an emotional state, such as anger or sadness), *restrained eating* (restricting food intake due to weight concerns), and *external eating* (eating because of external cues, such as the sight or smell of delicious food). For various statements, such as for emotional eating “When I feel lonely, I would love to eat something”, the degree of agreement is recorded from 1 (‘never’) to 5 (‘very often’). Total scores for the three subscales will be calculated. Higher values indicate a stronger expression of the corresponding eating style. Overall, the German version of the DEBQ shows good psychometric properties [51].

#### Dietary behavior

For the purpose of the intervention, the dietary intake questions are closely linked to sub-behaviors targeted in the intervention. Therefore, a list of 12 food groups has been formulated. Participants will be asked to indicate the average intake frequency for a usual week ranging from 0 (‘never’) to 5 (‘every day’). Hereafter, the average portion per day has to be chosen from a list of predefined portions (in grams accompanied by real life examples) specific for each food group. The list consists of the following food groups: a) vegetables, b) fish, c) meat, d) meat replacement products/eggs, e) fruit, f) processed pasta/rice, g) potatoes/whole grain pasta/whole grain rice, h) sweet and savory snacks, i) seeds and nuts j) whole grain bread, k) white bread, l) sugared drinks (fruit juice/sugar sweetened soft drinks).

#### Physical activity

Physical activity behavior will be assessed using the German short version of the International Physical Activity Questionnaire [52]. This questionnaire was developed to gather data about weekly physical activity of adults between the ages of 15 and 69 and shows acceptable psychometric properties [53]. In seven items, participants are asked to self-assess how many days out of the last seven days they did vigorous, moderate or walking activities and on those days how many hours and minutes they spent on each particular physical activity level. Time spent sedentary on a weekday is assessed as hours per day.

#### Depressive symptoms

Parental depressive symptoms will be assessed using the German version of the Edinburgh-Postnatal-Depression-Scale [54]. The EPDS is a 10-item self-report questionnaire for the screening of postpartum depressive symptoms in mothers and fathers [55, 56]. Items are rated on a 4-point-Likert scale ranging from 0 to 3. The overall score ranges from 0 (‘no postpartum depression’) to 30 (‘severe postpartum deprerssion’). Cox et al. (1987) report satisfactory psychometric properties.

#### Emotion regulation

Emotion regulation will be assessed through the short form of the Difficulties in Emotion Regulation Scale (DERS-18) [57, 58]. The questionnaire contains six subscales (*nonacceptance of emotional responses, difficulty engaging in Goal-directed behavior, impulse control difficulties, lack of emotional awareness, limited access to emotion regulation strategies and lack of emotional clarity*) with two to four items each (18 in total) that are rated on a 5 point-Likert scale ranging from 1 (‘almost never’) to 5 (‘almost always’). Overall, the DERS-SF showed good psychometric properties [59].

#### Experiences in close relationships

To assess partnership quality, the German version of the Experiences in Close Relationships-Revised (ECR-R) questionnaire will be used [60]. It is a 9-item short version to assess attachment anxiety and avoidance in adult partnerships. Participants rate statements concerning their partnership on a 7-point Likert scale ranging from 1 (‘strongly disagree’) to 7 (‘strongly agree’). Overall, the ECR-R has good psychometric properties [60].

#### Self-Efficacy

General self-efficacy will be assessed by the German version of the General Self-Efficacy Scale (GSE) [61]. The GSE assesses general optimistic self-beliefs to cope with difficult situations. It consists of 10 items, asking for one’s competence to cope with a range of challenging situations (e.g., ‘I can always manage to solve difficult problems if I try hard enough’). Responses to each item are given on a 4-point scale ranging from 1 (‘not at all true’) to 4 (‘exactly true’). To obtain a score for perceived self-efficacy the answers to the single items are summed up into one score. The GSE shows acceptable psychometric properties [62].

#### Parental childhood maltreatment

The Childhood Trauma Screener (CTS) is the German short form of the Childhood Trauma Questionnaire (CTQ) with five items [63, 64]. The CTS will be used to assess parental childhood maltreatment experiences. Participants rate emotional, physical, and sexual abuse as well as emotional and physical neglect on a 5-point-Likert scale ranging from 1 (‘not at all’) to 5 (‘very frequently’). Based on a validation study, high adversity was defined by scores higher than 10 [65].

#### Parenting stress

The German Version of the Parenting Stress Index – Short Form [66, 67] will assess the parental stress during the postpartum period. The self-report questionnaire contains 28 items with seven subscales. Each subscale includes four items rated on a 5-point Likert-scale ranging from 1 (‘strongly disagree’) to 5 (‘strongly agree’) with higher scores indicating higher levels of stress. Studies showed that the psychometric properties are good [66–69].

#### Parent-Child interaction

Parent-child interaction will be assessed by the German version of the Postpartum Bonding Questionnaire [70]. Originally, the questionnaire was developed as a screening instrument in order to diagnose bonding disorders consisting of 25 items yielding scores on four factors [71]. The German version consists of 16 items. The response categories range from 0 (‘always’) to 5 (‘never’) on a 6-point Likert scale with a higher sum score indicating impaired bonding. The sum scores range between 0 and 80 points. Reck et al. [70] report satisfying psychometric properties.

#### Parental perception of infants’ emotions

The IFEEL pictures (Infant Facial Expressions of Emotion from Looking at Pictures) ([72] have been employed previously in parents at risk for obesity and psychosocial stress to measure their ability to recognize children’s emotions [73, 74]. Six positive, six negative and four ambiguous pictures will be included in the current study, identified by Liel et al. [74] . Pictures are categorized as positive (negative) if rated as positive (negative) by at least 70% of the participants [75]. The internal consistency of positive/negative IFEEL Pictures was excellent for mothers and fathers [74].

#### Regulatory disorders

To gauge the potential stress by a present regulatory disorder, families will be asked to rate how burdened they are by the crying, sleeping and eating behavior of their child. For each of these three areas, families will answer one question, each on a 4-point Likert scale and ranging from 0 (‘not at all’) to 4 (‘very’). For eating behavior – since this is a special focus of this study, two additional questions will be asked that measure diagnostic criteria of an undereating disorder [76].

#### App usability

In order to assess app usability the German version of the mHealth app usability questionnaire for standalone mHealth apps (MAUQ; patient version) will be used [46, 77]. The questionnaire consists of 18 items, which are rated on a scale from 1 (‘strongly disagree’) to 7 (‘strongly agree’). Psychometric properties were rated as very good [46, 77]. The MAUQ has a three-factor design comprising the subscales ‘ease of use’ (5 items), ‘interface and satisfaction’ (7 items), and ‘usefulness’ (6 items).

#### App usage

The quantitative (i.e., login, logout times, sessions and modules completed, time spent in the app) and qualitative (i.e., participants input, used features, content utilized) app usage and engagement will be assessed by the apps’ meta-data log files. The fundamental data are internally tracked within the app and downloaded to secure servers. Treatment adherence will be operationalized as time spent using the app, numbers of days using the app and worked-through sessions and modules. If variance of data allows for it, we will define user patterns (e.g., heavy vs. minimal users) as outcome and mediator variables.

### Data collection and management

Data will be collected through three (Germany) or four (Austria) online assessments. Participating mothers and fathers complete the questionnaires separately, so that we will receive at least one questionnaire per family (from the mother) for every time point. Links to the assessments are sent out to the families at the corresponding time points (see the description of the procedure above for more details). The questionnaires should ideally be completed in the days after the family receives the link and not later than one month after that. If this period is exceeded at t1, participants will be excluded from this measurement point, but will still receive invitations to later time points. Furthermore, there will be two short online surveys for the HPs gathering information about their professional background and the families’ exclusion and inclusion criteria. We will also collect app-usage data from participants of the intervention arm. The data will be kept in accordance with the data protection guidelines of the European Union. Contact details will be maintained separately from questionnaire data. The collected data will only be marked with the individual study codes. The list with codes will be kept separately and is password protected.

### Blinding

For reasons inherent to the type of the interventions, participants cannot be blinded for the intervention. Families will be told about the group assignment of the HPs during the recruitment process. Statistical analyses will be performed blinded for allocation.

### Statistical analyses

In multilevel analyses, we will assess whether the clustered nature of the data (individuals clustered within families and HPs) has an effect on the results. If present, the impact of the intervention on the primary outcome and secondary outcomes will be analyzed using multi-level-models. If not, linear regression analysis will be used. Analyses of intervention effects will be based on the intention-to-treat principle. Given the cluster randomized design of the study, analyses on intervention effects will be adjusted for baseline values of the outcome. If suitable, missing data will be checked for randomness and be imputed using multiple imputations. Furthermore, we plan to assess the influence of moderating (stressful life events, parent child interaction, social support, partnership quality) and mediating (emotion regulation, self-efficacy) variables to identify determinants and pathways for the intervention’s efficacy and unhealthy weight gain during pregnancy and postpartum.

## Discussion

The transition to parenthood represents a crucial phase for the development of psychosocial stress and overweight in parents and their offspring. Families with high psychosocial burden are considered as a vulnerable group who are often not reached through existing preventive measures [22]. Therefore, I-PREGNO with its focus on this group fills an important gap. Perinatal mHealth interventions have the potential to overcome this problem and provide a low cost and low threshold intervention that promotes the mental and physical health of psychosocially burdened families. In this research project, we developed a blended counseling intervention named I-PREGNO that combines a face-to-face counseling program with a self-guided app. I-PREGNO consists of a comprehensive prevention strategy that addresses psychological transdiagnostic mechanisms underlying not only unhealthy weight gain, but also a wider range of mental health problems. The intervention supports families throughout the complete perinatal period and provides specialized content for both pregnancy and the postnatal period. In addition, we use a family-based approach, which means that we have created a version for mothers and a version for fathers, addressing the needs of both parents separately. During the developmental phase of the intervention, we used an iterative user-centered design with a participative approach to increase the ease of use and the fit to the future user’s needs (i.e., psychosocially burdened families). Furthermore, we collaborated with HP of a German *Early Childhood Intervention* program to facilitate the implementation of our intervention in the current care structures. Furthermore, the results of the present study will not only provide further evidence for the efficacy of mhealth-based interventions, but will also contribute information about a vulnerable and neglected group, psychosocially burdened families, and provide more information about their burdens, risk and protective factors, needs, and acceptance of preventive interventions.

While there are already many smartphone-based interventions for pregnancy and the postpartum period, I-PREGNO is the first mHealth intervention that targets psychosocially burdened families. With the present study, we aim to examine the efficacy of I-PREGNO on unhealthy weight gain and families’ psychosocial stress within a large, multinational cRCT of vulnerable families during pregnancy and the postpartum period. A major challenge of our study will be to reach the calculated sample size, as the target group is difficult to reach. In order to minimize recruitment problems, we established contacts with coordinators of institutions and HPs throughout Germany and Austria who have experience with the target group and who will consult us during the recruitment. We plan to recruit mothers and fathers and aim to generate more evidence for the efficacy of smartphone-based interventions and to gain more knowledge about the acceptance of digital interventions within psychosocially burdened samples. As we are already in contact with stakeholders and policy institutions, our intervention could easily be translated in other languages and transferred to public health policy.

## Data Availability

Not applicable.

## Abbreviations

BMI: Body Mass Index
cRCT: Cluster randomized controlled trial
CTS: Childhood Trauma Screener
DEBQ: Dutch Eating Behaviour Questionnaire
DERS-SF: Difficulties in Emotion Regulation Scale
ECR-RD: Experiences in Close Relationships
EPDS: Edinburgh-Postnatal-Depression-Scale
FFQ: Food Frequency Questionnaire
GSE: General Self-Efficacy Scale
HP: Healthcare professional(s)
IPAQ: International Physical Activity Questionnaire
MAUQ: mHealth app usability questionnaire
mHealth: Mobile health
PBQ: Postpartum Bonding Questionnaire
PSI-SF: Parenting Stress Index
SES: Socioeconomic status
TAU: Treatment as ususal
